# One-Step Synthesized Folic Acid-Based Carbon Dots: A Biocompatible Nanomaterial for the Treatment of Bacterial Infections in Lung Pathologies

**DOI:** 10.3390/nano15211657

**Published:** 2025-10-30

**Authors:** Gennaro Longobardo, Francesca Della Sala, Giuseppe Marino, Marco Barretta, Mario Forte, Rubina Paradiso, Giorgia Borriello, Assunta Borzacchiello

**Affiliations:** 1 Institute of Polymers, Composites and Biomaterials, National Research Council (IPCB-CNR), Viale J.F. Kennedy 54, 80125 Naples, Italy; gennaro.longobardo@unina.it (G.L.);; 2 Department of Chemical, Materials and Production Engineering, University of Naples Federico II, Piazzale V. Tecchio 80, 80125 Naples, Italy; 3 Department of Environmental, Biological and Pharmaceutical Sciences and Technologies (DiSTABiF), University of Campania “L. Vanvitelli”, 81100 Caserta, Italy; 4 Department of Experimental Medicine, University of Campania “L. Vanvitelli”, 81100 Caserta, Italy; mario.forte1@studenti.unicampania.it; 5 Istituto Zooprofilattico Sperimentale del Mezzogiorno, Via Salute, 2, 80055 Portici, Italy; rubina.paradiso@izsmportici.it (R.P.); giorgia.borriello@izsmportici.it (G.B.)

**Keywords:** folic acid, carbon dots, antimicrobial activity, lung infection

## Abstract

Bacterial infections are a major complication in chronic obstructive pulmonary disease (COPD) and acute respiratory distress syndrome (ARDS), where mucus accumulation and pH fluctuations further hinder treatment. Nanostructured systems such as carbon dots (CDs) are increasingly investigated as antimicrobial agents due to their scalability, low cost, and biocompatibility, compared to conventional antibiotics. Here, CDs were synthesized by a one-step microwave-assisted method at three reaction temperatures (130 °C, 170 °C, and 185 °C, named LT-CDs, MT-CDs, HT-CDs, respectively) to explore the effect of carbonization on their structure and function. TEM, Raman, and FTIR analyses were employed to investigate the size and distribution of carbon groups. UV–vis confirmed distinct pH-dependent spectral responses, and mucoadhesion studies revealed stronger and more stable interactions for MT-CDs. Biological assays demonstrated high biocompatibility across all samples on lung fibroblasts, while antimicrobial tests highlighted a selective effect against Staphylococcus aureus, due to ROS generation. Overall, MT-CDs represented the best compromise in terms of size, functionalization, biocompatibility, mucoadhesion, and antimicrobial activity, emerging as promising nanoplatforms for respiratory infection management in COPD and ARDS.

## 1. Introduction

Bacterial infections pose a significant challenge in the realm of respiratory diseases, especially when facing chronic obstructive pulmonary disease (COPD) and acute respiratory distress syndrome (ARDS) [1]. As a matter of fact, microbiota has been seen changing when contracting COPD, and these infections can also exacerbate the underlying conditions, leading to heightened morbidity and mortality rates [2]. COPD represents a chronic pathology, progressive and partially reversible airflow obstruction, with lung hyperinflation and significant extra pulmonary manifestations, mainly caused by long-term exposure to harmful gases or particles [3]. Here, bacterial infections can play a pivotal role in COPD exacerbations, leading to accelerating impairment of lung functions. It has been reported that acute exacerbation of chronic obstructive pulmonary disease was responsible for 1.5 million emergency department visits, 726,000 hospitalizations, and approximately 119,000 deaths in 2000 [4,5]. Conversely, ARDS has proven to be one of the major clinical problems in respiratory medicine. Even though its definition is ever-changing due to different and contrasting outcome data, it is characterized by sudden and severe respiratory failure, often precipitated by various underlying conditions such as pneumonia, sepsis, or trauma [6,7]. In the context of COPD and ARDS, bacterial infections commonly involve pathogens such as *Staphylococcus aureus* and *Pseudomonas aeruginosa* [8,9,10]. These infections further compromise lung function, exacerbating the already precarious respiratory status of affected individuals. Addressing the problem of bacterial infections in ARDS and COPD necessitates a multifaceted approach, especially in terms of an appropriate antimicrobial therapy. This antimicrobial therapy is crucial, especially due to the increasing problem of multidrug resistance (MDR) in microorganisms, mainly due to overconsumption of antibiotics and their broad utilization in agriculture, as well as the lack of novel antibiotics [11]. In order to overcome MDR effects, the use of nanostructured systems has gained increased attention lately, due to their scalability, low cost, versatility [12], and higher biocompatibility in contrast to the potential side effects associated with the misuse of traditional antibiotics [13]. Moreover, they offer novel antimicrobial modalities to fight bacteria by surprising bacterial defense mechanisms, providing new opportunities to overcome the effects of MDR and to eradicate pulmonary infections [14]. Notably, as a specific category of nanomaterials, carbon dots (CDs) are a promising class of carbon-based materials with several properties, such as biocompatibility, low toxicity, low cost, and ease of functionalization [15,16]. More importantly, CDs have shown intrinsic broad-spectrum antibacterial activity, mainly due to their ability to generate reactive oxygen species (ROS), which can kill bacteria [17]. However, their effects vary widely depending on their specific chemistry, size, doping, and functionalization [18]. CDs can act through multiple mechanisms, such as adhering to bacterial surfaces, disrupting nutrient access and causing leakage of intracellular contents, and binding to bacterial DNA [19]. To date, many studies use folic acid (FA) as a precursor for FA-CDs synthesized via hydrothermal or pyrolytic methods. With these synthetic strategies, the properties of the CDs depend solely on the precursor chemistry and offer less tunability in terms of structure–function relationships [20,21]. This is because they are focused on fluorescence, biocompatibility [22], molecular detection, and imaging for theranostics and cancer therapy applications for the delivery of chemotherapeutic agents [23]. Indeed, FA-CDs offer an additional dimension of targeting capability due to the folic acid moiety, which can bind to folate receptors overexpressed in certain cells (e.g., tumors or inflamed tissues) [24,25]. In contrast, microwave-assisted synthesis allows precise control of the degree of carbonization by adjusting reaction parameters (such as time or temperature) [26,27,28], enabling the production of CDs with customized size, surface chemistry, and pH-dependent optical behavior, better suited for antimicrobial applications in the respiratory environment. Although antibacterial applications based on FA-CDs have been reported in the literature, they are often achieved through a synergistic effect with antimicrobial agents such as drugs (e.g., curcumin, gentamicin) [29,30] or metals (Ag, Cu) [31,32], setting aside the specific optimization of these systems, which possess potential intrinsic antimicrobial properties for respiratory applications. Within this framework, and with the aim of producing nanostructured materials with intrinsic antimicrobial properties, CDs have been developed using FA as the main precursor. FA has been selected as a natural compound not only for its excellent biocompatibility but also for its rich chemical structure. Indeed, with the aim to synthesize CDs with intrinsic antimicrobial properties, FA is an ideal precursor, since it contains functional groups such as amines, carboxyls, and hydroxyl groups, which partially remain on the surface of the CDs after synthesis, making them highly reactive and easily dispersible in biological environments and favoring interaction with bacterial membranes [30,33]. Furthermore, the presence of nitrogen in the FA structure can integrate into the CDs, enhancing ROS production [18]. Moreover, the use of FA fits into a green chemistry approach, as it is a sustainable, non-toxic, and readily available precursor [20], enabling the synthesis of nanostructured materials without the use of hazardous reagents, contributing to a more environmentally friendly synthesis. In this work, FA-CDs were synthesized by a bottom-up approach using a simple and rapid one-step microwave-assisted system, with the aim of investigating different temperature settings (130 °C, 170 °C, and 185 °C). The resulting FA-CD synthesis was consistent with a green approach, as it used a renewable and biocompatible source (folic acid) as a precursor and exploited microwave irradiation to reduce reaction times, energy consumption, and the use of toxic solvents, minimizing the environmental impact of the process [34]. Thus, the physicochemical properties of FA-CDs, and consequently the functional properties in terms of biological and antimicrobial responses, were studied under varying synthesis temperatures. To this end, the physicochemical characterization (TEM, dynamic light scattering, Fourier transform infrared spectroscopy, Raman spectroscopy, thermogravimetric analysis, UV–vis absorbance) of FA-CDs was performed to evaluate their structure, such as N-doping efficacy and absorbance properties. Eventually, biocompatibility (i.e., Alamar blue assay and cell morphology) was evaluated on human lung fibroblast cells, and the percentage bacterial growth on *P. aeruginosa* and *S*. *aureus* was assessed, along with the FA-CDs’ ability to produce ROS. Moreover, FA-CDs’ mucoadhesive properties were evaluated at different pHs by studying their interaction with mucin via UV–vis absorbance. Overall, the results demonstrated that varying the synthesis temperature of FA-CDs affected key parameters influencing their functional properties. In particular, the temperature of 170 °C represented the best compromise capable of tailoring the structural properties of nanomaterials to address lung antimicrobial infections.

## 2. Materials and Methods

### 2.1. Microwave-Assisted Synthesis, Purification, and Yield

FA-CDs were prepared using FA and urea (Sigma, St. Louis, MO, USA) as precursors by the one-step microwave-assisted method. Firstly, 200 mg of FA and 200 mg of urea were dissolved in 10 mL of distilled water (pH = 7) and added in a 10–20 mL glass reaction tube to form the precursor solution. After 15 min ultra-sonication at 59 Hz, the reaction tube was put into a Biotage Initiator+ microwave synthesizer (Biotage, Uppsala, Sweden) for a fixed reaction time t = 10 min, with magnetic stirring equal to 600 rpm, under different reaction temperatures, T = 130°, 170°, and 185 °C (Table 1). FA-CDs were named, respectively, low-temperature CDs (LT-CDs), medium-temperature CDs (MT-CDs) and high-temperature CDs (HT-CDs). Once reacted, the system changed color, changing from a yellowish turbid to a brownish clear solution. The suspension was preliminarily centrifuged at 13.000 rpm for 15 min, in order to eliminate eventual carbonization residues. The supernatant was withdrawn and furtherly dialyzed (0.5–1 kDa MWCO) for four days in water, in order to dispose of possible unreacted precursors, and then the dialyzed product was freeze-dried. Reaction weight yield was estimated using Equation (1), as a ratio between the mass of precursors and the mass of the obtained CDs after the purification process, and was used to optimize reaction parameters:

(1) $$Yield\;\left(\%\right)=\frac{m_{FA}+m_{Urea}}{m_{CDs}}\cdot 100$$

### 2.2. Dimensional Distribution and Zeta Potential at Different pHs

Transmission electron microscopy (TEM, FEI Tecnai G12 Spirit Twin, FEI Company, Hillsboro, OR, USA) was used to investigate the variations in dimensional distribution (i.e., size and polydispersity index) and morphology as a function of the synthesis temperature of the precursor solution. Briefly, investigation was performed with an emission source LaB6 (120 kV, spot size 1) using a 400-mesh carbon-coated copper grids at room temperature (RT). The carbon-coated copper grid was immersed in a 5 mg/mL aqueous FA-CDs suspension in NaOH 1 mM, and, after the drying phase, the grid was placed on a rod holder for TEM characterization. Three grids per FA-CDs suspension were prepared, and a minimum of four micrographs per grid were acquired. Images of FA-CDs were imported into ImageJ software (v 1.54e, NIH, Bethesda, MD, USA) for postprocessing analysis and diameter quantification. Zeta potential (ζ) measurements were performed using a Zetasizer Nano (Malvern Instruments, Malvern, Worcestershire, UK) in order to evaluate the effect of synthesis temperature and pH (i.e., 5, 6 7, 8) on the FA-CDs’ stability. The different pHs were obtained starting from two solutions, including the most alkaline and the most acidic one, and then diluting them with Milli-Q water to adjust to the other pH values. This was performed to avoid acid–base neutralization, which could have changed the ionic strength of the solution, interfering with the measurement [35]. The concentration of FA-CDs dispersions was 5 mg/mL at a set temperature of 25 °C. Then, the Smoluchowski approximation was assumed to convert electrophoretic mobility into ζ potential values.

### 2.3. Fourier Transform Infrared (FT-IR) and Raman Spectroscopy

Aiming to preliminarily investigate the formation of CDs in terms of rearrangement of molecular bonds, FA-CDs obtained from different reaction protocols were characterized using PerkinElmer Frontier Fourier transform infrared spectroscopy FT-IR (Waltham, MA, USA), with a single-reflection universal ATR-IR accessory. All spectra were recorded between 4000 and 650 cm^−1^, with a resolution of 4 cm^−1^. Then, Raman spectra of FA-CDs were collected by a confocal Raman spectrometer (Horiba-Jobin Yvon Mod. Labspec Aramis, Edison, NJ, USA). The, samples were excited using a diode laser excitation source emitting at 632, 532, and 488 nm, with a spectral resolution < 1.5 cm^−1^. The 180° back-scattered radiation was collected by an Olympus metallurgical objective (MPlan 50×, NA  =  0.75), and the baseline spectra were initially corrected with a third order polynomial and normalized to the maximum peak intensity.

### 2.4. Thermogravimetric Analysis (TGA) and Derivative Thermogravimetry (DTG) of Precursors and FA-CDs

TGA was preliminarily performed on the precursors (i.e., FA and urea) to study their stability in the range of reaction temperatures investigated and was subsequently extended to the synthesized FA-CDs in order to highlight the important phases of weight loss. The experiments were conducted using Pt sample holders and approximately 7 mg of FA, urea, and CDs. A temperature ramp was set from 25 to 700 °C, with a nominal heating rate of 4 K/min under nitrogen flow for precursor analysis. Additionally, in order to identify potential structural differences among differently synthesized FA-CDs, a TGA/DTG was also performed on the CDs. Therefore, a temperature ramp was set from 40 to 1000 °C, with a nominal heating rate of 10 K/min under nitrogen flow.

### 2.5. UV–Vis Absorbance as a Function of pH

In order to investigate the effect of size distribution on quantum confinement effects, ultraviolet–visible (UV–vis) absorption spectra were measured. FA-CDs (33 μg/mL) were dispersed in ultrapure water, and UV–vis spectra were recorded using a PerkinElmer Lambda 900 spectrophotometer in a standard 10 mm quartz cuvette. The analysis was conducted in the 200–500 wavelength range, with 1 nm spectral resolution, 1 nm/s acquisition rate, and 2 nm slit settings. In order to evaluate the effect of environmental pH on the optical properties, which are strictly related to surface functionalization, the absorbance peak values at 280 nm were reported as a function of pH. Data were fitted with a second order polynomial, which was used to extract the differential absorbance dependence on pH (i.e., $\frac{dAbs}{dpH}$ vs. pH).

### 2.6. Biological Properties

#### 2.6.1. Cell Culture and Biocompatibility Assay

The HLF (Primary Lung Fibroblast, Normal, Human) cell line was grown in a T-75 cell culture flask (Falcon, Corning Inc., Corning, NY, USA) in complete Fibroblast Growth Kit-Low Serum, supplemented with rh FGF b, (0.5 mL, 5 ng/mL), L-glutamine (18.75 mL, 7.5 mM), ascorbic acid (0.5 mL, 50 µg/mL), hydrocortisone hemisuccinate (0.5 mL, 1 µg/mL), rh insulin (0.5 mL, 5 µg/mL), and FBS (10.0 mL, 2%), in a humidified and controlled atmosphere at 37 °C and 5% CO_2_. The medium was changed every 3–4 days. The cells were detached after confluent growth was reached, with 0.25% trypsin–EDTA solution and washed twice with PBS. The resulting cell suspensions were centrifuged (5 min, 1200 rpm; BRK55/10 Centrifuge by Centurion Scientific Ltd., Chichester, West Sussex, UK), the supernatant separated, and the cells re-suspended in a fresh culture medium. Viable cells were counted using the TC20 automated Cell Counter (Biorad, Hercules, CA, USA). In order to understand the in vitro biocompatibility of FA-CDs obtained at different synthesis temperatures, the cells were seeded at a density of 2 × 10^3^ cells/mL on 96-wells (Sigma-Aldrich, St. Louis, MO, USA), with each condition tested in triplicate. After 24 h, the culture media containing FA-CDs synthesized at different temperatures were incubated at concentrations of 50, 125, 250, 500, and 1000 µg/mL, for various incubation times (1, 3, 5, and 7 days of culture). Then, the Alamar blue assay (AB) was performed by adding AB reagent to the samples (at 10% *v*/*v* relative to the culture medium) and incubated at 37 °C for 4 h. The absorbance of the samples was measured using a spectrophotometer plate reader (Multilabel Counter, 1420 Victor, Perkin Elmer, Waltham, MA, USA) at 570 nm and 600 nm. AB is an indicator dye that incorporates an oxidation–reduction indicator that changes color in response to the chemical reduction in the growth medium, resulting from cell viability. Cells seeded in cell media without CDs were used as a control. Data are expressed as the percentage difference between treated and control groups to evaluate the percentage of reduction (*Reduction* %), which is calculated with the following formula (Equation (2)):

(2) $$Reduction(\%)=\frac{\left(O_{2}\times A_{1}\right)-(O_{1}\times A_{2})}{\left(O_{2}\times P_{1}\right)-(O_{1}\times P_{2})}\times 100$$

where *O*1 is the molar extinction coefficient (*E*) of oxidized AB at 570 nm; *O*2 is the *E* of oxidized AB at 600 nm; *A*1 is the absorbance of test wells at 570 nm; *A*2 is the absorbance of test wells at 600 nm; *P*1 is the absorbance of control well at 570 nm; and *P*2 is the absorbance of the control well at 600 nm. The percentage of reduction for each sample was normalized to the percentage of reduction for the control to obtain the cell viability percentage [36].

#### 2.6.2. Morphology of HLF Cells

Fluorescence microscopy was further used to observe HLF morphology after 7 days of incubation with CDs. In order to remove deposited CDs, the samples were washed with PBS (1×), three times, fixed with 10% formalin (Sigma-Aldrich, St. Louis, MO, USA) for 3 h, and permeabilized with 0.1% Triton X-100 for 3–5 min. The actin filaments were stained with TRITC phalloidin for 30 min at RT. Then, cell nuclei were stained with 4′, 6-diamidino-2-phenylindole DAPI, (Sigma Aldrich, St. Louis, MO, USA) for 10 min at 37 °C after two washes with PBS. Samples were observed by JuLI^TM^ Stage microscopy (NanoEntek, Waltham, MA, USA) with a 20× objective. Images were acquired with a resolution of 1024 × 1024 pixels.

### 2.7. Antimicrobial Activity

Overnight cultures of *Staphylococcus aureus* (SA) and *Pseudomonas aeruginosa* (PA) grown in Brain Heart Infusion Broth (BHI, Oxid) at an incubation temperature of 37 °C were used to prepare bacterial cell suspension for antibacterial activity tests. Bacterial cells were washed twice with saline solution and adjusted to a turbidity level equal to 0.5 McFarland standard (BioLife, Milan, Italy). The bacterial growth measurement was performed in 96-well plates. Each well contained 20 μL of bacterial cell suspension and various concentrations (7500, 3750, 1875, 938, 469, and 234 μg/mL) of CDs obtained by the different protocols in the BHI medium. The treated bacterial samples were then incubated at 35 °C for 24 h. The absence of growth at a given dilution was identified as the minimum inhibitory concentration (MIC) value. The analysis was performed in triplicate. The optical densities (OD) of the samples were measured at a wavelength of 600 nm after incubation using Multiskan FC Microplate Photometer (ThermoFisher Scientific, Waltham, MA, USA). The percentage of bacterial growth was calculated by divided the OD of each samples for the OD of control untreated group. The percentage (%) of inhibition was calculated according to the literature [37] using Equation (3):

(3) $$\%\;inhibition\;=\frac{AbControl(+)-Abtest\;agent}{AbControl(+)+AbControl(-)}\times 100$$

### 2.8. ROS Generation by L-Ascorbic Acid Absorbance Reduction

To evaluate the oxidase-like activity of FA-CDs synthesized at different temperatures in terms of reactive oxygen species (ROS) generation, the progressive decrease of ascorbic acid (AA) at its absorption maximum of 266 nm after its oxidation to dehydroascorbic acid was monitored. Briefly, an AA working solution was prepared in PBS 1 × (0.2 mM). Then, 100 μL of AA solution were dissolved in PBS 1 × as a positive control, in H_2_O_2_ 10 mM as a negative control, and in LT, MT, and HT FA-CDs (at concentrations of 17.5 and 50 μg/mL) to a final volume of 2 mL. The solutions were incubated at 37 °C under gentle stirring for 4 h, then UV–vis absorbance spectra were recorded using a PerkinElmer Lambda 900 spectrophotometer in a standard 10 mm quartz cuvette. Analysis was conducted in the 200–450 wavelength range, with 1 nm spectral resolution, 1 nm/s acquisition rate, and 1 nm slit settings. Eventually, the absorption peak reduction for each sample was normalized to the positive control (i.e., PBS 1 ×) absorbance peak value to calculate the percentage of absorbance.

### 2.9. FA-CDs Interaction with Mucin by UV–Vis Absorption and FTIR

The mucoadhesive capacity of the samples was evaluated by turbidimetric measurements using porcine stomach mucin (Type II, Sigma, Darmstadt, Germany) at a fixed concentration of 1.25 mg mL^−1^ [38]. Then, LT-CDs, MT-CDs, and HT-CDs were tested under different pH conditions (2, 4, 6, 7, 8, and 10) to assess the influence of medium acidity/alkalinity on mucin–nanoparticle interactions. For each pH, stock solutions of mucin were prepared at different pH values and then mixed with the concentrated CD solution to obtain mixtures at a concentration of 33 µg/mL. The control samples consisted of individual CD and mucin solutions prepared under the same pH conditions. To ensure homogeneity, all solutions were kept in the dark at RT under constant magnetic stirring before mixing. Polymer/mucin mixtures were incubated under magnetic stirring at RT for 1 h, 3 h, and 5 h, and each condition was tested in triplicate. The absorbances of CD suspensions ([*Abs*]*_CDs_*) and mucin ([*Abs*]*_mucin_*_)_ were recorded as controls. The theoretical absorbance of a non-interacting system ([*Abs*]*_theoretical_*) was calculated as the algebraic sum of the individual components, according to following Equation (4):

(4) $${\left[Abs\right]}_{theoretical}={\left[Abs\right]}_{CDs}+[Abs]mucin\;$$

The measured absorbance of the mixtures [*Abs*]*_exp_* was then compared with the theoretical value. An increase in [*Abs*]*_exp_* relative to [*Abs*]*_theoretical_* indicated the occurrence of interaction. The absorbance difference Δ[*Abs*], representing the interaction extent, was calculated according to Equation (5):

(5) $$\Delta [Abs]={\left[Abs\right]}_{exp}-{\left[Abs\right]}_{theoretical}\;$$

Moreover, in order to study their interaction with mucin, FA-CDs obtained from different reaction protocols were lyophilized in a 1:20 ratio to the mucin mixture and characterized by FTIR, as described previously.

## 3. Results and Discussion

### 3.1. Microwave-Assisted FA-CDs Synthesis

**Figure 1:**
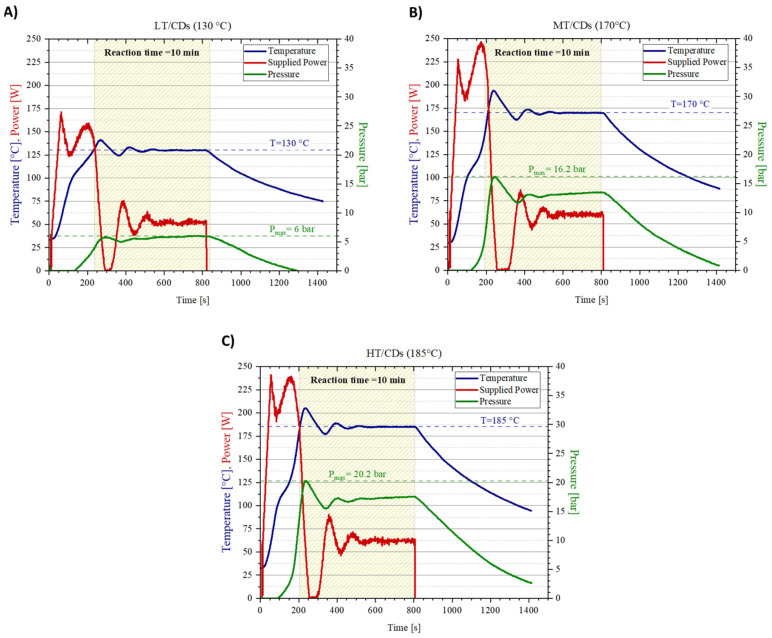
Synthesis protocol plots for CDs at different reaction temperatures. Temperature (in blue), supplied power (in red), and pressure (in green) monitored during reactions for CDs synthesized at pH = 7 under conditions of 130 °C (**A**), 170 °C (**B**), and 185 °C (**C**).

To conduct a microwave synthesis of the nanomaterials under pressurized conditions and to precisely monitor reaction parameters (i.e., supplied power, temperature increase, and pressure in the reaction vessel), the Biotage Initiator+ microwave synthesizer was used. As a matter of fact, this specific reaction system shows all the advantages of microwave-assisted synthesis (direct heating of target molecules, faster reaction times [39]), with some additional features overcoming the most common synthesis by domestic microwave irradiation: excellent reproducibility, higher yields, and purer compounds, with extremely precise and controllable size distribution [40]. The reaction plots are depicted in Figure 1, correlating the power supplied by the synthesizer with the temperature read by the thermocouple in the reaction vessel, as well as the pressure inside the chamber. Notably, the pressure is supposed to affect the reaction yield, since chemical reactions are more likely to occur under high pressure [41]. In this reaction configuration even lower temperatures than those employed in conventional microwave-assisted synthesis can be used, which may directly lower the risk of precursor’s degradation and, therefore, increase reaction yield. When T stabilizes for the MT and HT samples, the conversion of ammonium cyanate to gaseous ammonia in the urea degradation pathway can be one of the causes of the continuously increasing pressure of the system. As a matter of fact, pressure averagely stabilizes when reacting at T = 130 °C, but it does not reach the same plateau at higher temperatures because the system is continuously generating ammonia, progressively increasing the pressure in the chamber (apart from the contribution that increasing temperature brings to the pressure increase). By the end of the synthesis, it was possible to evaluate the mass yield. MT-CDs recorded the highest mass yield of 62 ± 5%, while LT and HT-CDs shared a similar mass yield of 53 ± 4% and 52 ± 4%, respectively.

### 3.2. Dimensional Distribution and Zeta Potential as a Function of pH

**Figure 2:**
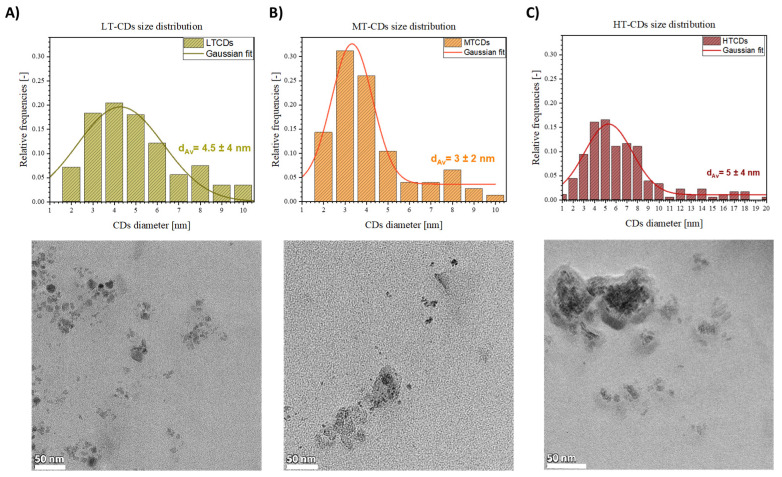
TEM acquisitions and dimensional analysis of CDs obtained at 130 °C (**A**), 170 °C (**B**), and 185 °C (**C**). Scale bars: 50 nm.

The TEM images in Figure 2 show the morphological features and size distribution of LT, MT, and HT-CDs. The size distribution histograms show a temperature-dependent trend, highlighting shifts in the size distribution based on the synthesis temperature used. Briefly, at low temperatures, the CDs appeared as quasi-spherical nanoparticles, with a relatively broad size distribution and an average diameter of 4.5 ± 4 nm. Increasing the synthesis temperature to medium conditions resulted in equally larger but more monodisperse particles, with an average size of 3 ± 2 nm. CDs with these dimensional characteristics have shown high biocompatibility, especially if derived from biological precursors such as folic acid; good electronic confinement that can increase the efficiency of electron or energy transfer, favoring the production of ROS; and greater penetration into bacterial membranes, favoring the interaction with the bacterial peptidoglycan and the membrane due to the large specific surface area [42,43]. At high temperatures, the CDs displayed a further increase in average size to 5 ± 4 nm, along with the presence of aggregated structures, likely due to higher kinetic energy promoting particle coalescence [44]. The overall behavior is consistent with thermally driven nucleation–growth mechanisms, where higher temperatures favor both the fusion of smaller nuclei and the formation of larger graphitic domains. Additionally, the increased aggregation observed in HT-CDs may be attributed to the reduced surface passivation efficiency at elevated temperatures, leading to stronger interparticle interactions.

**Figure 3:**
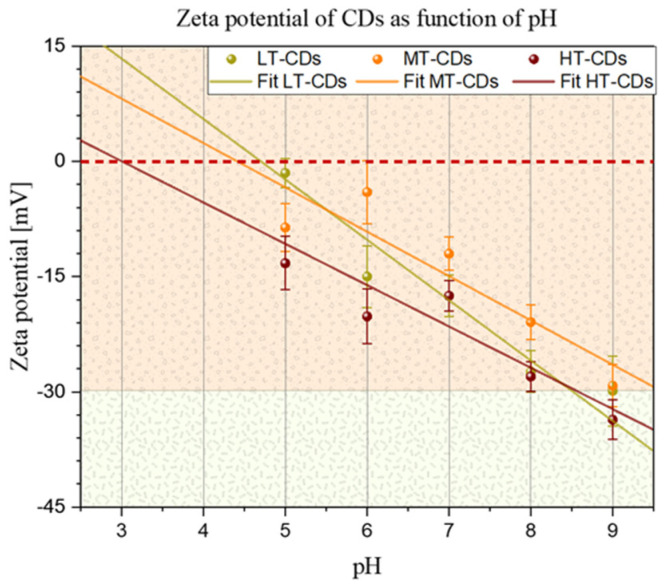
Zeta potential of CDs as a function of pH. Solid lines represent linear fits for all samples. Points in which the fitted lines cross the dashed red line represent the isoelectric point for each sample (i.e., 4.7, 4.4, and 3.0 for LT-CDs, MT-CDS, and HT-CDs, respectively).

To gain insight into the surface charge of CDs and their stability, the zeta potential of the different CDs was measured after they were suspended in different pH solutions (Figure 3), as the presence of ions has a strong effect on the surface charge of many types of particles. At neutral pH, the zeta potential was negative for all samples, with values of −17~3 mV for LT-CDs, −12~2 mV for MT-CDs, and −18~2 mV for HT-CDs, showing how the doping of two electron-rich groups on the surface influences the surface charge [23]. In particular, it is influenced by N-rich groups, such as –NH_2_ on the surface and abundant oxygen-containing groups, such as –OH and –COOH. The similarity in the zeta values falling roughly within the same range and within the limits of experimental uncertainty is attributed to the fact that all CDs share the common feature of hydroxyl and carbonyl groups heavily decorating the surface. However, the difference in the isoelectric point (i.e., IEP, the pH value at which ζ = 0) of the different CD suspensions seems to highlight a structural difference among the samples in terms of the functional groups decorating the surface. The difference in the IEP for the three samples is related to a different distribution of surface groups on CDs. The IEP value depends on the nature and quantity of ionizable groups on the surface of CDs: carboxyl groups (–COOH) deprotonate, conferring a negative charge; hydroxyl groups (–OH) are weakly acidic; while amino groups (–NH_2_) protonate, conferring a positive charge [45]. LT-CDs, MT-CDS, and HT-CDs showed values of 4.7, 4.4, and 3, respectively. These results indicated that the IEP decreases with increasing synthesis temperature, suggesting that HT-CDs have a more acidic surface, i.e., are richer in deprotonable groups (–COOH, –OH) and have fewer basic groups (–NH_2_). LT-CD (IEP = 4.7) would possess both more and less oxidized amino groups because the lower temperature better preserves the original bonds of the precursor (e.g., folic acid). Finally, MT-CD (IEP = 4.4) undergoes more pronounced carbonization, with a balance between carboxyl and amino groups. These data suggest that at acid pH values, the LT-CD and MT-CD will be close to their IEP and therefore less stable than the HT-CD, which will maintain its stability features, while at physiological pH values, stability increases with increasing synthesis temperature [38].

### 3.3. Fourier Transform Infrared (FT-IR) and Raman Spectroscopy

**Figure 4:**
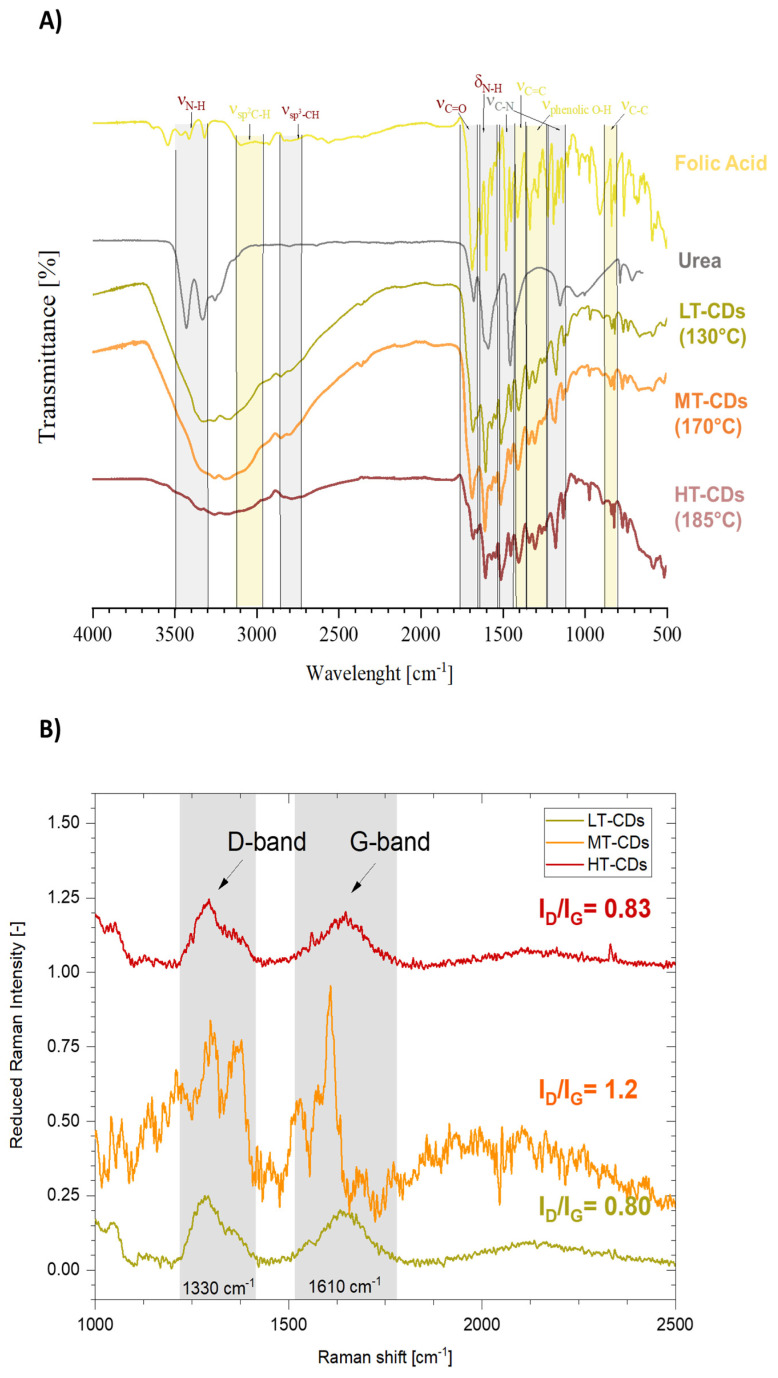
Fourier transform infrared (FT-IR) analysis of FA and urea precursors, as well as of CDs synthesized at different reaction temperatures (namely, LT-CDs, MT-CDs, and HT-CDs). Peaks deriving from precursors are highlighted in green, those strongly characteristic of CDs are represented in grey (**A**). Normalized Raman spectra of CDs: characteristic graphitic and disordered domain peaks from C-containing structures (I_G_ and I_D,_ respectively) are highlighted, and their ratio is reported (**B**).

The FT-IR analysis allowed us to assume some structural peculiarity of CDs when compared to the precursors (Figure 4A). FA showed a quite complex spectrum, especially when compared to urea, which showed two more resolved peaks between 3450 and 3300 cm^−1^, corresponding to the symmetric and asymmetric stretching of the N-H bond, attributed to amine groups. These peaks seem preserved in the LT-CDs but are apparently absent in MT and HT-CDs, where the wide zone going from 3400 to 2600 cm^−1^ is characteristic of high water retention in those samples. In addition, C=O stretching is stronger in FA and shows a stronger peak at 1686 cm^−1^ and a weaker one at 1667 cm^−1^ in the fingerprint region, due to the high presence of this kind of C=O bond along the structure in carboxyl groups and aromatic structures. In particular, when dealing with CDs, the only peak is the one at 1676 cm^−1^, due to the presence of carboxyl groups on the outer surface of CDs. Other representative peaks are those at 1602 and 680 cm^−1^, representative of bending and wagging vibrations of the amine group NH_2_, which are shifted to 1589 cm^−1^ in the CD samples. It can be observed that for the LT, MT, and HT samples those peaks progressively decrease in intensity due to the fact that the amine groups decorating the outer shell of CDs decrease in number as the CDs become bigger in size at higher synthesis temperatures [46,47]. Eventually, the presence of different peaks for C-N stretching in the 1510–1410 cm^−1^ and 1310–1210 cm^−1^ ranges is representative of different heterocyclic structures in CDs, possibly due to the presence of C-N bonds in amine groups, as well as graphitic and pyridinic N [48]. In the end, the slight variation in intensity and position of the C=C bond around 1400 cm^−1^ might represent the formation of graphitic structures in CDs due to carbonization reactions. Moreover, Raman spectroscopy was employed in Figure 4B to investigate the structural features of LT-, MT-, and HT-CDs, focusing on the relative intensity of the disorder-induced D band (ca. 1350 cm^−1^) and the graphitic G band (ca. 1580 cm^−1^) [49]. The I_D_/I_G_ intensity ratio provides valuable information on the balance between amorphous/defect domains and ordered graphitic regions [23]. Indeed, the D band is linked to structural imperfections in the carbon framework and is associated with the presence of sp^3^-hybridized carbon, which typically arises from the amorphous graphite formed during the oxidation process. In contrast, the G band originates from vibrations within the sp^2^-carbon atomic plane. A lower intensity ratio of the D to G bands (I_D_/I_G_) indicates better CD formation. This corresponds to a reduced amount of sp^3^ carbon, greater graphitization, and a uniform surface structure [23]. Studies have shown that doped CDs can also exhibit an I_D_/I_G_ ratio close to 1, as their structure is heavily influenced by their chemical composition, including both the surface termination groups and the dopant elements [50,51,52]. Notably, for all samples, the I_D_/I_G_ ratios fall within the reported range for high-quality carbon dots [53]. LT-CDs and HT-CDs exhibited relatively low I_D_/I_G_ ratios (0.80 and 0.83, respectively), indicating the presence of a partially ordered structure with some defects, which influence the *sp*2 hybridization pattern in the carbon structure and indicate the presence of hybrid groups on the surface [49]. While MT-CDs possess an I_D_/I_G_ ratio of 1.20, showing a more disordered structure, with defects associated with higher doping levels and an increased number of functional groups. Therefore, this may be related to the enhanced presence of active sites capable of interacting with biomolecules, enhancing antimicrobial efficacy [54]. Overall, MT-CDs exhibit an optimal balance between oxidation and nitrogen-doping that finely tunes their surface chemistry and electronic structure. Controlled oxidation introduces oxygen-containing groups that create defect sites and enhance surface reactivity, while preserving the conjugated carbon framework. Concurrently, nitrogen atoms, mainly in pyrrolic and pyridinic configurations, are incorporated within or at the edges of the carbon lattice, increasing electron density, passivating surface traps, and narrowing the bandgap. This balance has been proven to show an optimal radiative recombination, with an improved water solubility and chemical stability [55,56]. For this reason, MT-CDs are expected to have improved functional performance in biological environments, combining enhanced biocompatibility with effective antimicrobial activity [57].

### 3.4. TGA/DTG of Precursors and CDs

**Figure 5:**
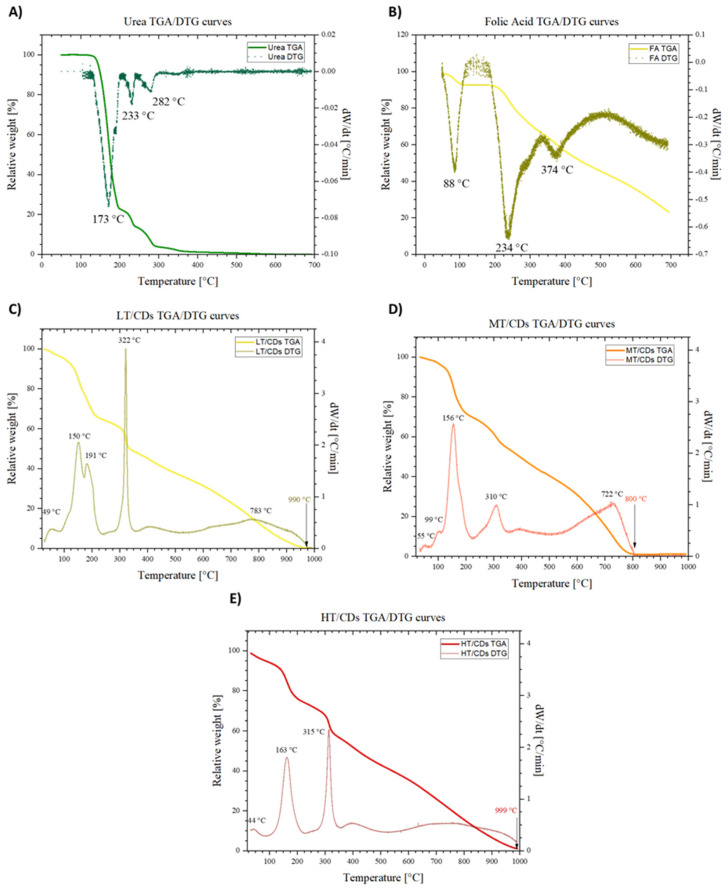
TGA and DTG curves of folic acid (**A**) and urea (**B**) in the temperature range from 25 to 700 °C. TGA and DTG curves of LT-CDs (**C**), MT-CDs (**D**), and HT-CDs (**E**) in the temperature range from 40 to 1000 °C. DTG peaks are highlighted, with the characteristic peaks representing degradation temperatures.

The TGA and DTG analyses allowed us to understand the thermal degradative pathways of FA and urea precursors, which are fundamental for understanding the synthesis process. In particular, urea showed a well-resolved degradation path, where a first degradative region, RT to ~190 °C, is associated with the decomposition of urea into different products, such as biuret, which, between 190 and 250 °C, begins to decompose. Then, the third region, between 250 and ~360 °C, is characterized by the continued sublimation and decomposition of the remaining products, which are, to a good approximation, totally eliminated at ~400 °C, reaching the total loss of mass [58] (Figure 5A). On the other hand, FA does not completely degrade in the temperature window investigated. In addition, the decomposition can be summarized as occurring in three stages (Figure 5B). Firstly, around 100 °C, the loss of adsorbed water takes place, leaving behind the anhydrous sample, since this molecule can establish H bonds in several sites, binding water molecules. Then, the glutamic acid moiety is lost, and thereby degraded, around 234 °C. Then, FA becomes an amorphous mass around 374 °C [59]. Once synthesis has occurred, the degradation spectra in Figure 5C–E show some important differences, reflecting structural change in CDs depending on the synthesis temperature. The first difference that can be observed is the fact that in a wider temperature window, the behaviors of CDs differ from one another. However, in all the samples, the mass drop that ends at 1000 °C shows complete decomposition of CDs to char. More generally, it can be observed that nearly all samples exhibit some DTG peaks that resemble those of FA in terms of positions, but they are shifted towards lower temperatures. This suggests partial pre-degradation during microwave synthesis and can be attributed to the high-energy environment causing the breakdown of weaker bonds, forming intermediates and new functional groups. These structural features may reduce thermal stability, leading to earlier degradation. In particular, the LT-CDs showed a behavior more closely resembling that of FA, with the only difference being a broader temperature range, and complete degradation of the material around 990 °C. The overall similarity of the TGA profile of LT-CDs with that of FA makes the hypothesis of a non-complete FA degradation plausible, or at least it allows us to say that during the formation of CDs, reagents did not undergo severe structure-changing reactions. On the other hand, HT-CDs show a quite similar trend. Therefore, the explanation for a similar behavior in thermal stability of these samples can be explained by means of a higher degree of carbonization and aromaticity. As a matter of fact, the samples treated at higher temperatures exhibit a smaller mass loss in the decomposition range of 100–250 °C, revealing the efficient removal of oxygen-containing functional groups. In addition, the higher threshold to full degradation (around 1000 °C) is representative of a very stable carbonous structure in the material, suggesting a high level of carbonization [60]. On the other hand, MT-CDs degrade at lower temperatures than LT- and HT-CDs due to incomplete carbonization, which leaves them with many thermally unstable, oxygen- and nitrogen-rich groups and low-molecular-weight fragments. The DTG trace for MT-CDs shows significant mass loss around ~156 °C and ~310 °C, consistent with the decomposition of labile surface groups and small oligomers from precursors like urea and folic acid. In contrast, LT-CDs retain more stable precursor structures, while HT-CDs are more completely carbonized, resulting in a delayed onset of degradation for both.

### 3.5. UV–Vis Absorbance at Different pH Levels

**Figure 6:**
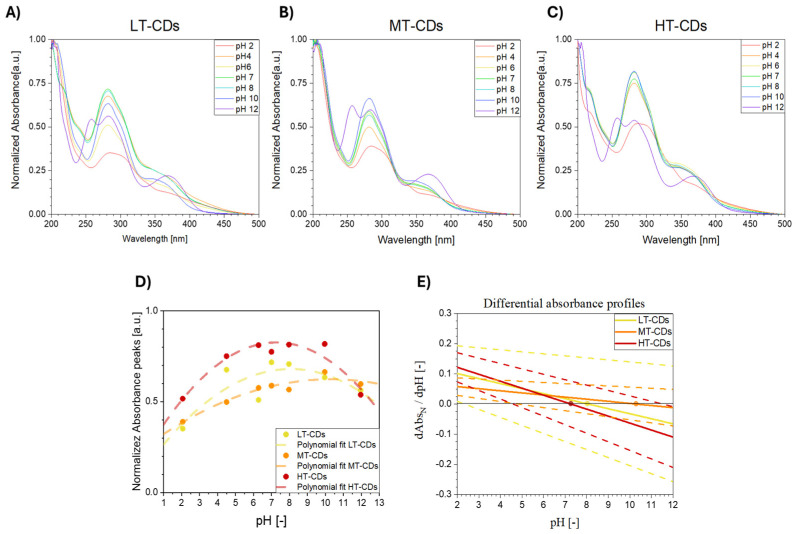
Normalized UV–vis absorbance spectra of LT-CDs (**A**), MT-CDs (**B**), and HT-CDs (**C**) in distilled water, at different pH values (2, 4, 6, 7, 8, 10, and 12), at a concentration of 33 μg/mL. Normalized absorbance peak intensity at 280 nm as a function of pH, with a polynomial fit represented by dashed lines (**D**), and differential absorbance profiles extracted from the polynomial fit; dots represent the inflection points, where the trend is inverted and dashed lines represent the uncertainties deriving from polynomial fit for each sample (**E**).

The UV–vis absorbance behavior of LT, MT, and HT-CDs was evaluated at different pH values, since it is highly sensitive to the protonation and deprotonation of surface functional groups and therefore provides an indirect evaluation of the different chemical environments and reactivity of CDs [61]. pH-dependent trends in both normalized and absolute spectra have been shown, particularly in the 280–350 nm region associated with π–π* (C=C) and n–π* (C=O, C–N) transitions. These spectral variations arise from protonation and deprotonation of surface functional groups such as –COOH, –OH, and –NH_2_, whose type, density, and distribution are influenced by synthesis conditions. In particular, protonation under acidic conditions typically results in a red shift and decreased absorbance intensity, while deprotonation under alkaline conditions can cause blue shifts or peak broadening. CDs with a high degree of oxidation or nitrogen doping often show more pronounced pH sensitivity due to the increased availability of ionizable groups [62,63]. LT-CDs showed modest spectral changes across the pH range, consistent with a high density of oxygenated groups and an amorphous structure, which together buffer the response and result in broad, less defined absorbance features (Figure 6A). MT-CDs displayed even less pronounced responses under mildly acidic and basic conditions, likely due to the emergence of a partial graphitic core coexisting with residual surface functionalities (Figure 6B). HT-CDs exhibited the most significant spectral shifts, along with lower absolute absorbance at pH 7, suggesting increased carbonization and a reduced—but more chemically responsive—surface group population (Figure 6C). However, when absolute absorbances are considered at acidic up to neutral pH values, they can be found to be higher for LT-CDs and MT-CDs than for HT-CDs, indicating that the latter, despite stronger relative responsiveness, may have lower light-harvesting capacity in neutral media. This is likely due to increased graphitization at high temperatures (Figure S1). A quantitative analysis via differential absorbance slopes $\frac{dAbs}{dpH}$ confirmed this trend: HT-CDs showed the steepest slope and highest pH sensitivity, while LT and MT-CDs displayed smaller, more gradual changes (Figure 6D). Overall, the results indicate that synthesis temperature modulates both the magnitude and sharpness of the optical response to pH. While LT- and MT-CDs offer higher absolute absorbance and smoother transitions, HT-CDs demonstrate greater responsiveness per unit absorbance, which may be advantageous for applications requiring high sensitivity to environmental pH. In particular, LT-CDs show relatively modest spectral changes across the pH range. This behavior is consistent with a high density of surface oxygenated functional groups (e.g., –COOH, –OH), which provide buffering capacity and lead to more gradual modulation of electronic transitions. The broad and less defined absorbance peaks further suggest an amorphous carbon structure with limited π-conjugation. In contrast, MT-CDs exhibit more pronounced spectral responses to pH changes, especially under mildly acidic and basic conditions. These dots likely represent an intermediate stage of carbonization, where a partial graphitic core begins to emerge, while retaining a significant number of surface functionalities. This dual character enhances their sensitivity to pH by enabling localized electronic transitions to be modulated by the chemical environment, as reflected by the increased normalized absorbance under basic conditions. HT-CDs, obtained at the highest synthesis temperature, demonstrate the most marked spectral variations across the pH spectrum. This heightened sensitivity can be attributed to a more defined sp^2^-hybridized carbon core and a reduced, but more reactive, population of surface groups, potentially including nitrogen dopants formed under high-temperature conditions. The strong modulation of the normalized pH in HT-CDs suggests that even small changes in the protonation state can induce significant shifts in electronic structure and transition probabilities. Overall, these features highlight the importance of synthesis conditions in tuning the optical response of CDs and suggest that MT-CDs are preferred when more stable properties are required at different pH values, such as in infection sites, while HT-CDs are particularly suitable for sensing applications where pH responsiveness is required [64].

### 3.6. Viability and Morphology of HLFs

**Figure 7:**
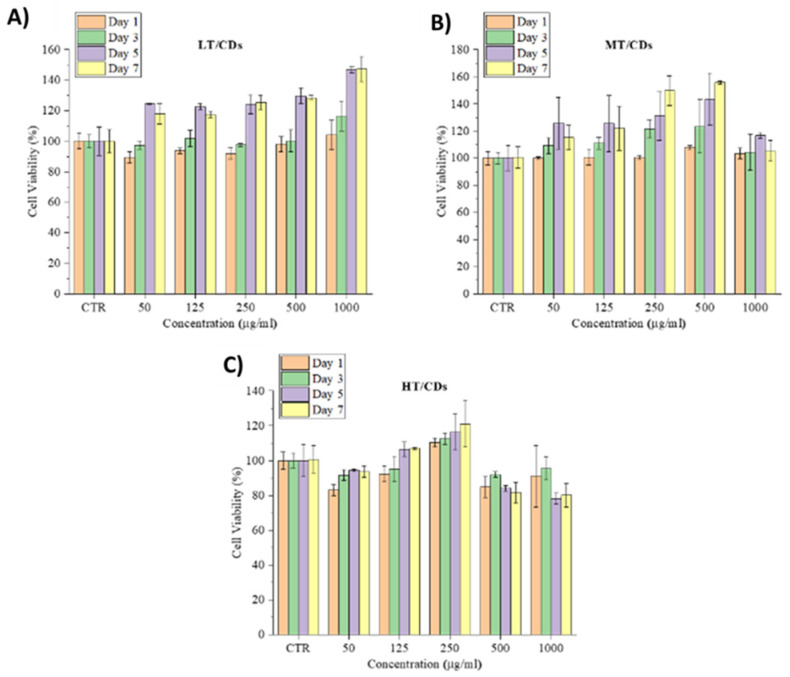
HLF cell viability seeded with different concentrations of LT (**A**), MT (**B**), and HT-CDs (**C**) after 1, 3, 5, and 7 days of culture.

Cell viability and morphological analyses demonstrated that FA-CDs are strongly biocompatible and do not induce cytotoxic effects in human lung fibroblasts (HLFs). Across all tested concentrations (50–1000 µg mL^−1^) and culture times (1, 3, 5, and 7 days), cell viability never fell below roughly 80%, a level widely accepted as fully biocompatible, and frequently exceeded the 100% baseline of untreated controls (Figure 7). A gradual, culture-time-dependent increase in viability was evident, reflecting a stimulatory, rather than inhibitory, response. In particular, the LT-CDs promoted the highest long-term viability, reaching around 120% on day 5 across all concentrations and increasing to approximately 140% at 1000 µg mL^−1^. MT-CDs exhibited a similar pattern, with their most pronounced effect at 500 µg mL^−1^ after 5 days, approaching 140%. This is the most biocompatible combination for which cell viability arises mostly. HT-CDs performed best at 250 µg mL^−1^; at higher concentrations a slight reduction was observed, possibly associated with the higher amount of carbonization products, but even these values remained close to or above the 80% safety threshold [65].

**Figure 8:**
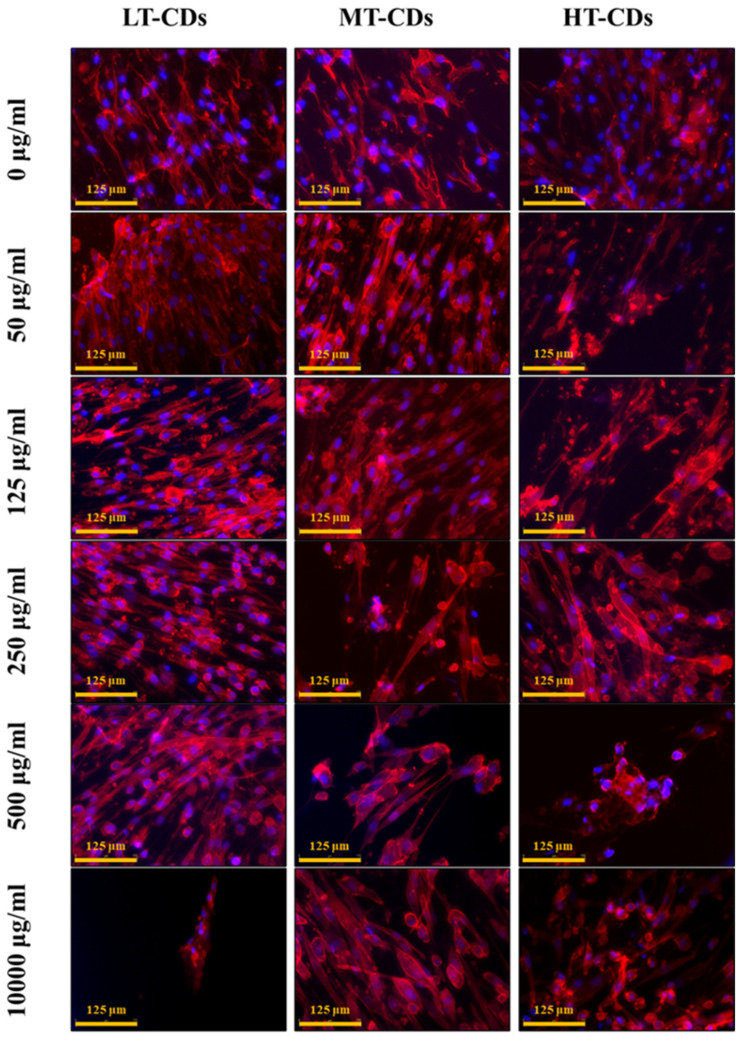
Morphological evaluation of HLF cells, assessed after 7 days of exposure to implemented media with different LT, MT, and HT-CDs-7 concentrations. Nuclei were stained with blue DAPI, and the actin filaments of the cytoskeleton were stained with red phalloidin. Scale bar: 50 µm.

Biocompatibility results were further confirmed by HLF cell morphology. Actin filaments, a constituent of the cytoskeleton, were stained with TRIC phalloidin after 7 days of incubation with systems. HLF cells, indeed, exhibited a typical, non-cytotoxic fibroblast-like morphology after incubation with the formulations at different concentrations (Figure 8). Their morphology was similar to the characteristic in vitro HLF morphology that is spread-shaped, as seen in control samples, often characterized by several extending processes, which consists of cell protrusions adhering to the flat surface. Overall, these results indicated that the nanodevices have extremely good biocompatibility also after 7 days of seeding. Thus, FA-CDs exhibited excellent biocompatibility, as they are derived from folic acid, a natural molecule that is essential for human metabolism. Indeed, the presence of residual functional groups (–NH_2_, –COOH, –OH) inherited from FA can enhance solubility and the interaction between the aqueous medium rich in nutrients and the cells, thereby reducing toxicity and promoting cell viability [25].

### 3.7. Antimicrobial Activity and Oxidase-like Activity by Monitoring ROS Generation

**Figure 9:**
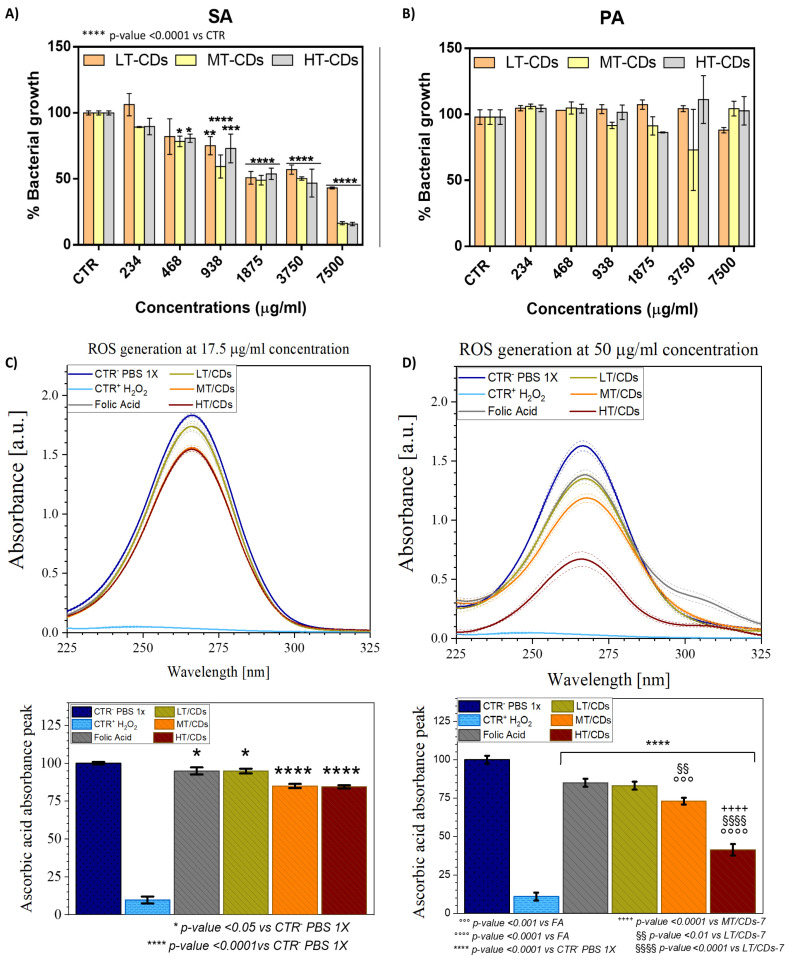
Percentage of bacterial growth of Gram-positive SA (**A**) and Gram-negative PA (**B**) after incubation with CDs at different concentrations. The percentage of bacterial growth was calculated relative to the non-treated bacteria, and the results are the mean of three independent experiments. * *p*-value < 0.05, ** *p*-value < 0.01, *** *p*-value < 0.001 vs. CTR. In the top panel ROS formation is shown via the reduction of the L-ascorbic acid (AA) absorbance peak (**C**,**D**). FA-CDs were tested at concentrations of 17.5 μg/mL (**C**) and 50 μg/mL (**D**). PBS 1X represents CTR+, while H_2_O_2_ represents CTR-. The percentage reduction in peak intensity is shown in the bottom graphs and linked to the oxidization degree of the molecule. All data are presented as mean ± standard deviation. Each error bar represents 1 standard deviation and serves as the estimate of standard uncertainty. The data are representative of 3 repeated experiments in triplicate (n = 3). */**** *p*-value < 0.05/0.0001 vs. CTR^-^ PBS 1x, °°°/°°°° *p*-value < 0.001/0.0001 vs. FA, ^++++^ *p*-value < 0.0001 vs. MT/CDs, ^§§^/^§§§§^ *p*-value < 0.01/0.0001 vs. LT/CDs.

The antimicrobial properties of synthesized LT-CDs, MT- CDs, and HT-CDs were tested against *Staphylococcus aureus* (SA, Gram-positive) and *Pseudomonas aeruginosa* (PA, Gram-negative), both clinically relevant in lung infections (Figure 9A,B). Concentrations ranged from 234 to 7500 μg/mL, and bacterial growth was expressed relative to untreated controls. CDs displayed selective activity against SA and a lack of inhibition on PA proliferation. As demonstrated in the literature, PA is notoriously difficult to treat. Being a Gram-negative bacterium, it possesses a highly effective outer membrane barrier, particularly rich in lipopolysaccharides (LPSs). LPSs are complex molecules that make the bacterial surface less permeable, limiting the entry of hydrophobic or electrically charged molecules, including many nanomaterials. Furthermore, PA is equipped with highly efficient efflux systems; these molecular “pumps” are able to actively expel toxic substances that manage to enter the cell. Among these, MexAB-OprM, one of the most studied, is capable of recognizing and removing a wide range of compounds, including antibiotics and potentially even nanoparticles such as carbon dots. This means that even if FACDs manage to partially penetrate the bacterial cell, they could still be rapidly eliminated [29,30]. For SA, growth reduction was detected at the lowest tested concentration of 234 μg/mL for MT- and HT-CDs (a growth % of 89.3 ± 0.4 and 89 ± 6%, respectively, corresponding to a bacterial inhibition % of ~11%), whereas LT-CDs required 938 μg/mL to produce measurable inhibition (growth % of 75 ± 7%, corresponding to an inhibition of ~25%). MT-CDs achieved the most pronounced effects, reducing bacterial growth by 59 ± 9% at 938 μg/mL (corresponding to ~40% bacterial inhibition) and 16 ± 1% (~85% inhibition) at 7500 μg/mL, matching HT-CDs at the highest concentration. The MIC values suggested that LT-CDs necessitated a high concentration of around 18 ± 6 mg/mL, while MT-CDs and HT-CDs of about 9.2 ± 0.8 mg/mL and 8.9 ± 0.6 mg/mL, respectively achieve comparable values. These data are corroborated from the analysis of Raman spectra. Indeed, as demonstrated, the I_D_/I_G_ ratio for all samples showed a good level of controlled defects (I_D_/I_G_ ratio around 0.8–1.2), which is reported to contribute to increasing the antimicrobial efficacy of these nanomaterials [49]. The presence of more defects, as in the case of the MT-CDs structure (1.2), is related to the increased density of surface active sites (edge, oxygenated and nitrogenous groups, and folic acid residues) that can interact with the bacterial membrane, generate reactive species, or promote adhesion. Conversely, a material that is too ordered could be less reactive (fewer active sites) and therefore less effective as an antimicrobial agent. Antimicrobial activity is certainly also influenced by the surface charge of the nanomaterials [66]. The results of the surface charge indicated that all the FA-CDs produced had a moderate negative charge (e.g., −10–30 mV), suggesting that the preferred antibacterial mechanism of action could be through ROS generation rather than direct interaction with bacterial membranes, which are also negatively charged. Moreover, surface charge values demonstrated good colloidal stability, resulting in less aggregation and greater dispersion of the nanomaterials, increasing their availability to act on bacteria. To investigate the antimicrobial properties, the oxidase-like activity of FA-CDs synthesized at different temperatures was assessed. The depletion of AA was monitored by UV–vis spectroscopy as an indirect measure of ROS generation. This method relies on the oxidation of AA to dehydroascorbic acid, which corresponds to a decrease in absorbance at 266 nm (Figure 9C,D). In particular, at a 17.5 μg/mL concentration, a slightly significant oxidation of AA (about 5%, corresponding to a normalized peak intensity equal to 94.8%) occurs when using FA or LT-CDs (*p*-value < 0.05 vs. CTR^+^), while a highly significant change in oxidation degree is observed when MT-CDs and HT-CDs are used, with a relative absorbance peak of ~85 and 84% (corresponding to an oxidation degree of ~15 and 16%, respectively, *p*-value < 0.0001 vs. CTR^+^). When the concentration is increased to 50 μg/mL, further differences are highlighted among the different CD samples. As a matter of fact, with the increasing concentration, FA still shows the lowest effect on AA oxidation (15 ± 2%) yet has a highly significant effect with respect to the positive control (*p*-value < 0.0001 vs. CTR^+^). A slightly higher oxidation degree of 17 ± 3%, although not statistically significant with respect to FA, is shown with LT-CDs (with a relative absorbance peak of ~83%). The greatest differences emerge when dealing with MT-CDs and HT-CDs. As a matter of fact, MT-CDs show an oxidation degree of 28 ± 2%, corresponding to a peak reduction around 72%, which results in a highly significant (*p*-value < 0.0001) effect compared to CTR^+^ and a very significant one to LT-CDs (*p*-value < 0.01). As for HT-CDs, the oxidation degree increases to a consistently significant value of 59 ± 4% (corresponding to a peak reduction around 41%, *p*-value < 0.0001 vs. CTR^+^, FA, LT-CDs and MT-CDs). In the literature, it is known that ROS generation can be influenced by pH; FA-based CDs can exhibit increased ROS production at acidic pH [67]. However, MT- and HT-CDs were able to produce a high dose of ROS, thanks to the N-doping of the structure, even at neutral pH values. This characteristic makes these systems ideal for the potential treatment of persistent chronic infections, in cases of exacerbations of severe lung diseases (such as COPD and ARDS), in which the pH at the site of infection appears to slightly increase the pH above the values observed in healthy airways (7.6–7.8) [68]. As reported in literature, several types of ROS can be produced by CDs, including superoxide anion (O_2_^−^), hydrogen peroxide (H_2_O_2_), and hydroxyl radicals (OH) [69,70,71]. In this case, ROS generation could occur primarily through oxidase-like activity [72]. The most plausible mechanism involves the reduction of molecular oxygen by the CDs, leading to the formation of superoxide anions and, subsequently, hydrogen peroxide via dismutation [73]. Furthermore, the results demonstrated that the synthesis temperature of FA-CDs (LT, MT, and HT) affected their ROS-generating capacity. Indeed, higher synthesis temperatures generally lead to increased graphitization and a greater abundance of electron-donating or redox-active surface groups, which can enhance electron transfer to oxygen and thereby increase ROS production [45]. ROS quantification showed that HT-CDs produced the highest ROS levels. However, their greater carbonization could limit their surface interaction, making them less effective in the real application. Conversely, the smaller size of MT-CDs and their high ROS generation could also facilitate the cellular internalization and then intracellular disruption by ROS. This dual-action potential likely underlies their superior efficacy against SA, demonstrating the importance of controlling synthesis temperature to fine-tune the physicochemical properties and antimicrobial performance of CDs.

### 3.8. FA-CDs Interaction with Mucin by UV–Vis Absorption and FTIR

**Figure 10:**
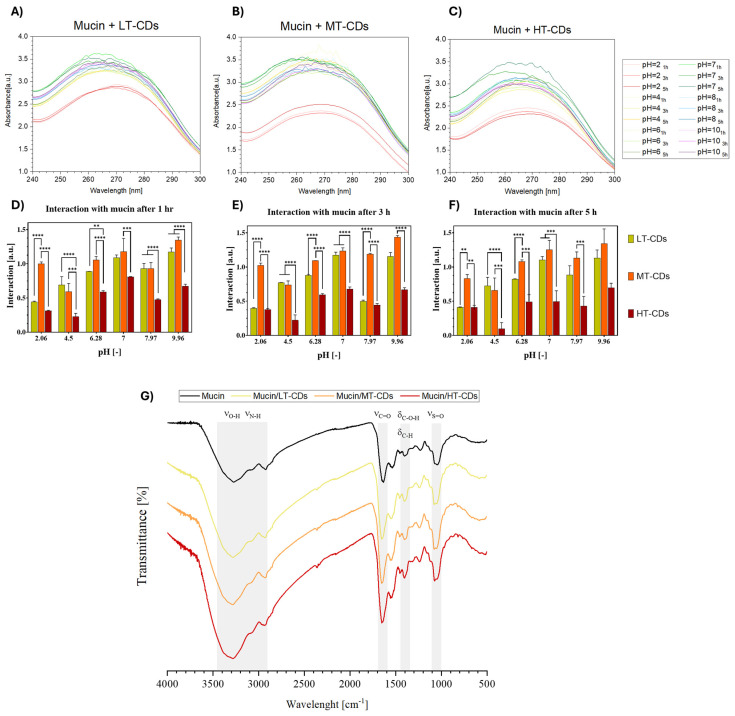
Mucoadhesion studies of LT-, MT-, and HT-CDs with mucin (1.25 mg mL^−1^) at different pH values (2, 4, 6, 7, 8, and 10) and contact times (1, 3, and 5 h). UV–vis absorbance spectra of mucin in the presence of LT-CDs (**A**), MT-CDs (**B**), and HT-CDs (**C**) at the indicated pH values and time points. Bar graphs reporting the calculated absorbance differences ΔAbs for LT-CDs, MT-CDs, and HT-CDs after 1 (**D**), 3 (**E**), and 5 h (**F**) of interaction with mucin under the different pH conditions. **** *p*-value < 0.0001, *** *p*-value < 0.001, ** *p*-value < 0.01. (**G**) FTIR spectra of mucin and mucin/FA-CDs complexes. Characteristic vibrational bands, corresponding to O–H/N–H stretching (νO–H, νN–H), amide I (νC=O), carbohydrate bending (δC–O–H, δC–H), and sulfate stretching (νS=O), are highlighted.

Mucosal tissues protect the human body by secreting mucus, whose major component, mucin, plays a central role in adhesion phenomena due to its cysteine-rich, high-molecular-weight glycoprotein structure [38]. Mucoadhesive properties could represent an advantage for nanomaterials designed to combat lung infections, especially in conditions such as COPD and ARDS. Indeed, mucoadhesiveness promotes greater retention and local targeting, which can improve antimicrobial efficacy and reduce side effects. Mucin is known to undergo intermolecular and hydrophobic interactions, which are pH-dependent and also affect UV–vis absorption properties (Figure S2). To investigate the mucoadhesive potential of the synthesized CDs, their interaction with 1.25 mg/mL^−1^ mucin solution was evaluated under different pH conditions (2, 4, 6, 7, 8, and 10) and contact times (1, 3, and 5 h). As shown in Figure 10, a clear pH-dependent trend was observed, with MT-CDs (Figure 10B) consistently displaying the strongest interaction with mucin across all conditions. This effect ranged from extremely significant differences (*p* < 0.0001) at acidic pH values to highly significant ones (*p* < 0.001) in more basic environments, particularly evident after prolonged contact (5 h). Regarding time-dependence, LT-CDs (Figure 10A) showed only minor, non-significant decreases in interaction over time, whereas HT-CDs (Figure 10C) exhibited a slightly more pronounced reduction, suggesting a loss of stability in mucin binding. By contrast, MT-CDs revealed a different trend, with interactions tending to remain stable or even showing slight, non-significant improvements with longer incubation, supporting their robustness as mucoadhesive systems. The overall results indicate that MT-CDs not only exhibit the highest interaction with mucin but also maintain it more effectively over time, with a tendency to increase or stabilize as the pH increases. These data are promising, considering that in the context of lung infections, local pH can vary depending on the pathology, e.g., active bacterial infection (5.6–6.2), [74], pneumonia (7.2–7.4) [75], rhinitis (7.2–8.3) [76], and chronic bronchitis (7.6–7.8) [77]. Moreover, these data indirectly indicate that in viscous microenvironments of the mucin (3 Pa s) (Figure S3), the increased interaction with mucin/MT-CDs could enhance the local production of ROS, especially at slightly alkaline pHs such as in chronically infected mucus. Thus, MT-CDs may represent the most promising candidates, as their mucoadhesive performance is maximized under such conditions. In order to characterize the interaction between mucin and FA-CDs, FTIR was performed (Figure 10G). The spectra of mucin and treated samples (mucin/LT-CDs, mucin/MT-CDs, and mucin/HT-CDs) revealed characteristic absorption bands associated with hydroxyl, amide, carbohydrate, and sulphate groups (Table S1) [78]. In the O–H/N–H stretching region (~3600–3000 cm^−1^), all samples exhibited a broad band at 3259, 3288, 3298, and 3282 cm^−1^ for mucin, LT, MT, and HT samples, respectively. The gradual shift toward higher wavenumbers upon treatment suggests a reduction in hydrogen bonding strength, possibly due to rearrangements in the hydration network or conformational changes in mucin glycoproteins. The amide I region (~1650 cm^−1^), primarily arising from C=O stretching vibrations of peptide bonds, showed peaks at 1635, 1652, 1660, and 1654 cm^−1^. The upward shift, most evident in mucin/MT-CDs, indicates protein backbone reorganization and alterations in secondary structure, consistent with partial unfolding or exposure of peptide domains. In the carbohydrate region (~1400 cm^−1^), minor shifts from 1400.38 to 1404.58 cm^−1^ were detected, reflecting modifications in glycosidic linkages or hydration states of the polysaccharide components. Finally, the sulfate region (~1240–1230 cm^−1^) exhibited characteristic S=O stretching bands at 1232, 1241, 1241, and 1240 cm^−1^, confirming the presence of sulphated glycoprotein moieties. The slight upshift in treated samples points to changes in sulphate exposure and electrostatic interactions, likely driven by structural rearrangement and hydration effects. Thus, the observed spectral variations highlight treatment-dependent modifications in the hydrogen bonding network, secondary structure, and glycosylation environment of mucin. Hence, these features lead to the observation that MT-CDs have a potentially prolonged residence time at inflamed sites, improving both therapeutic retention and local antimicrobial efficacy.

## 4. Conclusions

Bacterial infections remain a critical issue in respiratory diseases such as COPD and ARDS, where pH fluctuations and mucus accumulation complicate treatment. CDs offer novel antimicrobial strategies, surprising the bacterial defense systems and opening up new possibilities to bypass MDR in lung infections. In this study, FA-CDs were synthesized by a simple one-step microwave-assisted system at three different temperatures: LT-CDs (130 °C), MT-CDs (170 °C), and HT-CDs (185 °C), aiming to study the influence of synthesis temperature on the structural and functional properties of nanomaterials. The use of the Biotage Initiator+ microwave synthesizer enabled the synthesis of CDs under highly controlled, pressurized conditions, ensuring improved reproducibility. The ability to monitor the temperature, pressure, and power input in real time highlighted the crucial influence of these parameters on the reaction progress and final yield, with MT-CDs showing the highest conversion efficiency. TEM morphological analysis and surface properties of CDs highlighted a clear dependence on the temperature synthesis parameters, showing MT-CDs as the most balanced in terms of size, monodispersion, and functionalization, while HT-CDs exhibited aggregation tendencies and a more acidic surface. The FT-IR analysis showed a progressive decrease in amino groups and an enrichment in carboxyl groups with increasing temperature, suggesting greater surface oxidation and more severe carbonization in the MT and HT samples. Raman data confirmed these observations, showing a high I_D_/I_G_ ratio for the MT-CDs, indicative of greater functionalization. TGA/DTG showed that while LT-CDs exhibited thermal characteristics similar to the precursor, suggesting partial carbonization, HT-CDs exhibited superior thermal stability, indicative of a more aromatic and carbon-rich structure. MT-CDs, on the other hand, exhibited early degradation, linked to the presence of functional groups. The UV–vis analysis suggested that MT-CDs are preferred when more stable properties are required at different pHs, such as in infection sites. Cell viability tests and morphological analysis confirmed the high biocompatibility of FA-CDs with human lung fibroblasts, even at high concentrations and for prolonged periods, with LT- and MT-CDs showing the greatest stimulatory effects on cell viability. FA-CDs showed selective antimicrobial activity against *S. aureus*, while they were ineffective against *P. aeruginosa*, likely due to their structural barrier represented by the LPS-rich outer membrane and the efflux mechanisms. Among the different samples, MT-CDs stood out for their efficacy, with a significant reduction in bacterial growth even at low concentrations, thanks to an optimal balance between a size favorable for internalization and the ability to generate ROS. HT-CDs, despite showing the greatest production of ROS, could be less effective, likely due to their greater degree of carbonization, which may limit their surface interaction. Conversely, LT-CDs showed lower antimicrobial efficacy, consistent with their lower reactivity and oxidative capacity. Eventually, CDs exhibited differentiated interactions with mucin, with MT-CDs standing out for their higher and more stable adhesion to the mucosa, representing a significant advantage in the local retention time for applications against pulmonary infections, where the microenvironment is often inflamed and pH-altered. In conclusion, this study underscores the importance of synthesis temperature as a key factor in designing tunable CDs as antimicrobial nanomaterials. The overall results demonstrated that MT-CDs possessed the best balance of properties, such as size, stability, functionalization, biological activity, and greater effectiveness, against S. aureus, highlighting their potential for treating lung infections in COPD and ARDS diseases.

## Figures and Tables

**Table 1:** Synthesis protocols for different samples.

Sample	Reaction Temperature [°C]	Reaction Time [min]	Precursors Solution pH
LT-CDs	130	10	pH = 7 (Water)
MT-CDs	170	10	pH = 7 (Water)
HT-CDs	185	10	pH = 7 (Water)

## Data Availability

The data presented in this study are openly available in the article.

## Supplementary Materials

The following supporting information can be downloaded at https://www.mdpi.com/article/10.3390/nano15211657/s1. Figure S1: UV–vis absorbance spectra of LT-CDs (A), MT-CDs (B), and HT-CDs (C); Figure S2: UV–vis absorbance spectra of mucin at different pH values. Figure S3: Flow curve of a 1.25 mg/mL1 mucin solution. The viscosity η decreases markedly with increasing shear rate, indicating a pronounced shear-thinning behavior. Experimental data (blue circles) were fitted using the Cross model (redline), yielding a zero-shear viscosity η_0_ of approximately 5.32 Pa s and a flow index n around 0.23 in the power-law region. Table S1: FTIR band assignments and peak positions for mucin and treated samples. Refs. [47,67,79] are cited in the Supplementary Materials file.
