# Supplementary material for: One-Step Synthesized Folic Acid-Based Carbon Dots: A Biocompatible Nanomaterial for the Treatment of Bacterial Infections in Lung Pathologies
Source: Nanomaterials (Basel). 2025 Oct 30;15(21):1657. doi: 10.3390/nano15211657 (PMC12610706; doi:10.3390/nano15211657)
Supplement: Supplementary file 1 [file nanomaterials-15-01657-s001.zip › nanomaterials-3937577-supplementary.pdf]

Supporting information

One-Step Synthesized Folic Acid based-Carbon Dots: A Biocompatible Nanomaterials for Treating Bacterial Infections in lung pathologies

Gennaro Longobardo[1,2], Francesca Della Sala[1*], Giuseppe Marino[1,3], Marco Barretta[1,3], Mario Forte[4], Rubina Paradiso[5], Giorgia Borriello[5] and Assunta Borzacchiello[1*]

[1]Institute of Polymers, Composites and Biomaterials, National Research Council (IPCB-CNR), Viale J.F. Kennedy 54, 80125, Naples, Italy
[2]Department of Chemical, Materials and Production Engineering, University of Naples Federico II, Piazzale V. Tecchio 80, 80125, Naples, Italy
[3]Department of Environmental, Biological and Pharmaceutical Sciences and Technologies (DiSTABiF), University of Campania "L. Vanvitelli", 81100 Caserta, Italy
[4] University of Campania "L. Vanvitelli", 81100 Caserta, Italy
[5]Istituto Zooprofilattico Sperimentale del Mezzogiorno, Via Salute, 2, 80055 Portici, Italy

* correspondence to: Dr. Assunta Borzacchiello: bassunta@unina.it and Dr. Francesca Della Sala: francesca.dellasala@cnr.it

UV-Vis absorbance of CDs at different pHs

The UV/Vis absorbance spectra of LT-, MT-, and HT-CDs at different pH values (**Figure S1**) revealed clear trends linked to the degree of carbonization. In all cases, the main peak around 280 nm, associated with π–π* transitions of aromatic C=C bonds and n–π* transitions of C=O/C–N groups, showed pH-dependent modulation. LT-CDs (**Figure S1A**) displayed relatively small spectral changes across the pH range, consistent with a higher density of oxygenated functional groups that buffer protonation–deprotonation events and produce broad, less defined spectra. MT-CDs (**Figure S1**) exhibited even more stable behaviour, with less pronounced pH responsiveness likely due to a partial graphitic core coexisting with surface functionalities. Instead, HT-CDs (**Figure S1C**) showed the strongest modulation of absorbance with pH, reflecting a more carbonized sp^2 network with fewer but more reactive surface groups. Quantitative analysis of the 280 nm absorbance peak (**Figure S1D**) confirmed these observations: LT- and MT-CDs showed gradual slopes, while HT-CDs exhibited the steepest slope and thus the greatest pH sensitivity. Differential absorbance analysis further highlighted this trend, with HT-CDs being the most responsive system, albeit with lower absolute absorbance compared to LT- and MT-CDs. Overall, the results indicate that while LT- and MT-CDs maintain higher absorbance stability across pH values, with MT-CDs showing the best absorbance performance in all pH range, HT-CDs provide sharper, less stable optical responses.

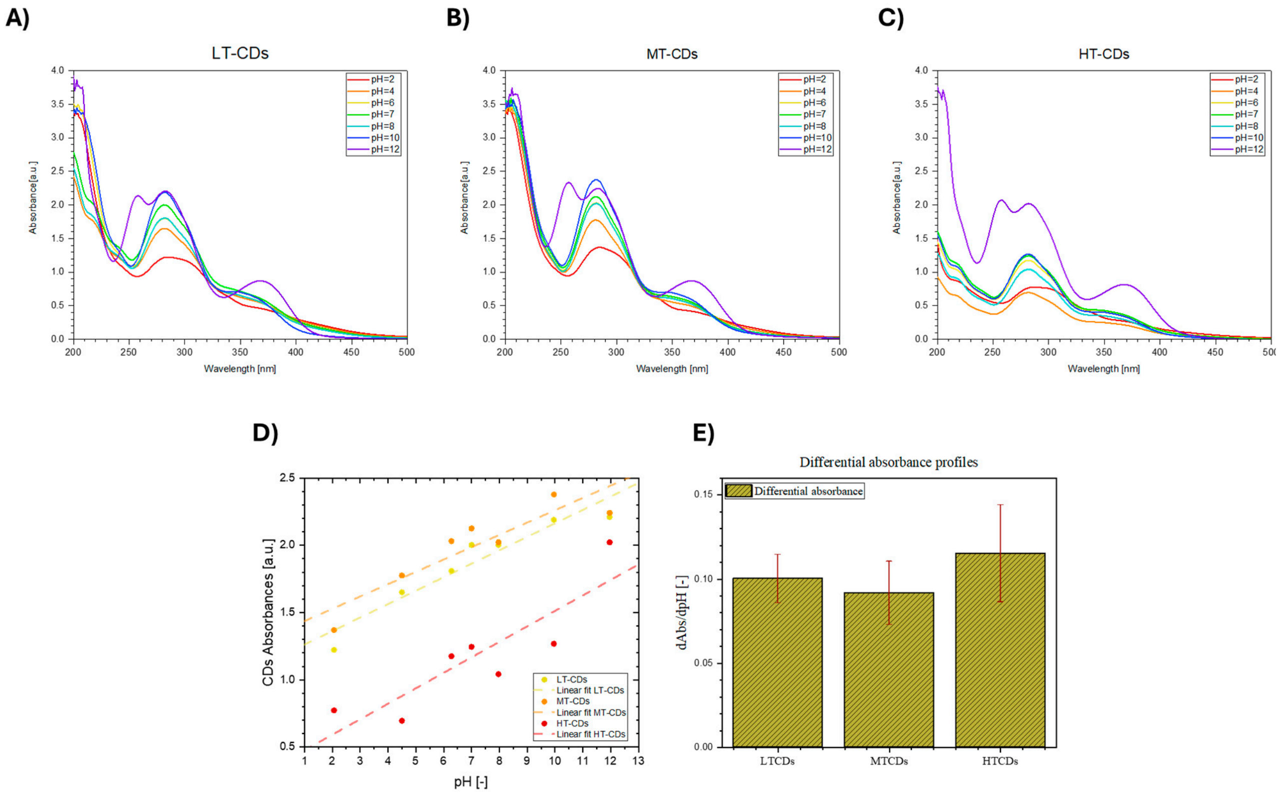

Figure S1. UV/Vis absorbance spectra of LT-CDs (**A**), MT-CDs (**B**) and HT-CDs (**C**) in distilled water at different pHs (2, 4, 6, 7, 8, 10 and 12), at a concentration of 33 μg/ml. 280 nm absorbance peak intensity as a function of pH with polynomial fit represented by dashed lines (**D**) and differential absorbance values extracted from linear fit (**E**).

UV-Vis absorbance of mucin at different pHs

The UV/Vis absorbance spectra of mucin was obtained at different pH values (2,4, 6, 7, 8 and 10) in distilled water and. Spectra reported in **Figure S2** show a broad absorption band around ~261 nm, which is typically attributed to aromatic amino acids and glycoprotein chromophores present in mucin. The absorbance intensity shows a clear dependence on the pH: at acidic conditions (pH 2), the spectrum displays the lowest intensity, suggesting partial conformational changes or reduced exposure of chromophoric groups. As the pH increases, particularly between neutral and slightly basic values (pH 7–8), a marked increase in absorbance is observed, indicating enhanced structural stability of mucin and greater exposure of aromatic residues. At alkaline conditions (i.e. pH 10), the signal remains elevated but slightly decreases compared to pH 7–8, which may reflect partial

rearrangements or destabilization of the glycoprotein backbone under strongly basic conditions. Overall, these results confirm that mucin maintains its most stable and optically active conformation under near-neutral to slightly basic environments, whereas acidic conditions induce significant spectral changes consistent with protein unfolding or aggregation. This pH-dependent behaviour was already seen to be cause of sol-gel transition at higher concentrations [3] and was particularly relevant for subsequent interaction studies with carbon dots, since it highlights the role of mucin's structural state in modulating potential bio-interactions with the nanosystem.

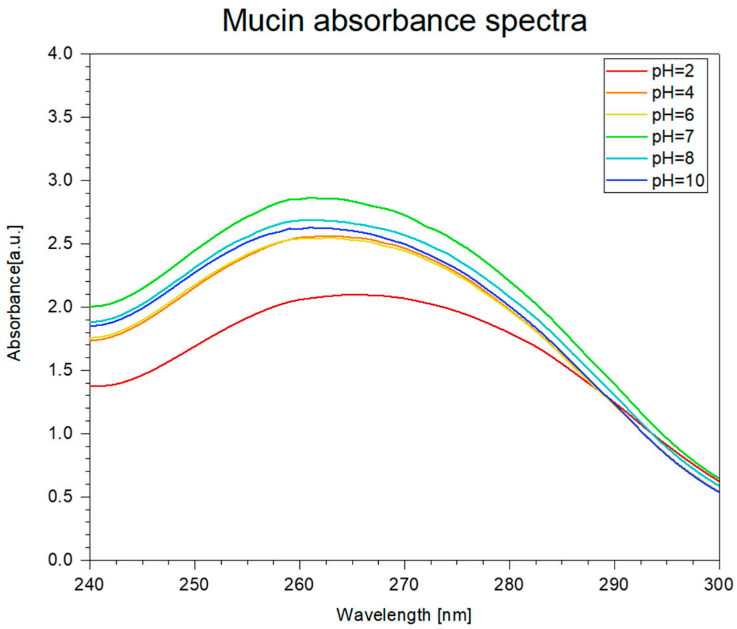

Figure S2. UV/Vis absorbance spectra of mucin at different pHs (2, 4, 6, 7, 8 and 10) at a concentration of 1.25 mg/ml.

FTIR peaks assignements for Mucin and Mucin-CDs

Region (assignment)	Vibrational mode	Mucin (cm^{-1})	Mucin_LT (cm^{-1})	Mucin_MT (cm^{-1})	Mucin_HT (cm^{-1})
ν O–H / N–H stretching	Hydrogen-bonded OH and amide A	3259.25	3287.96	3298.36	3282.36
ν Amide I	C=O stretching (peptide linkage)	1634.93	1652.43	1660.00	1653.83
δ Carbohydrate region	C–H and C–O–H bending (glycosidic linkages)	1400.38	1398.28	1397.58	1404.58
ν Sulfate region	S=O asymmetric stretching (sulfated glycoproteins)	1231.65	1240.75	1240.75	1240.05

Table S1. FTIR band assignments and peak positions for mucin and treated samples.

Rheological Characterization: viscosity of Mucin Solution

The rheological behavior of mucin solution was evaluated using a **Haake Mars III rheometer** (Thermo Fisher Scientific, Germany) equipped with a cone-plate geometry (diameter 35 mm, angle 1°). A mucin solution at a concentration of **1.25 mg/mL** was prepared in deionized water and allowed to hydrate overnight under gentle stirring. Flow curves were recorded at 25 °C by ramping the shear rate from 0.001 to 500 s^{-1}, and the corresponding viscosity values were collected. The measurements were performed in triplicate to ensure reproducibility. The experimental data were subsequently fitted using the Cross model to extract key rheological parameters, to describe the shear-thinning behavior of the mucin solution.

The flow curve for the 1.25 mg/mL mucin solution exhibits a characteristic shear-thinning behavior, spanning over three decades of viscosity. The viscosity (η) decreases sharply from a high value at the lowest measured shear rates around 3 Pa s. The viscosity subsequently transitions into an infinite-shear Newtonian plateau η_∞ around 0.001 Pa s for shear rates greater than 100s^{-1}. This significant

reduction in viscosity under increasing shear is typical for high-molecular-weight polymers like mucin, reflecting the progressive disentanglement and alignment of the polymer chains under flow.

The data were accurately fitted using the Cross model, which successfully captured the full range of the transition from the zero-shear to the infinite-shear regime. Further analysis of the shear-thinning region, performed using a power-law model, yielded a very low flow index n=0.22 ±0.01. Since n<<1, this confirms the highly non-Newtonian nature of the mucin solution and quantifies the high degree of shear-thinning. This rheological profile underscores the strong internal structuring and inter-chain interactions within the mucin network at rest and under low stress.

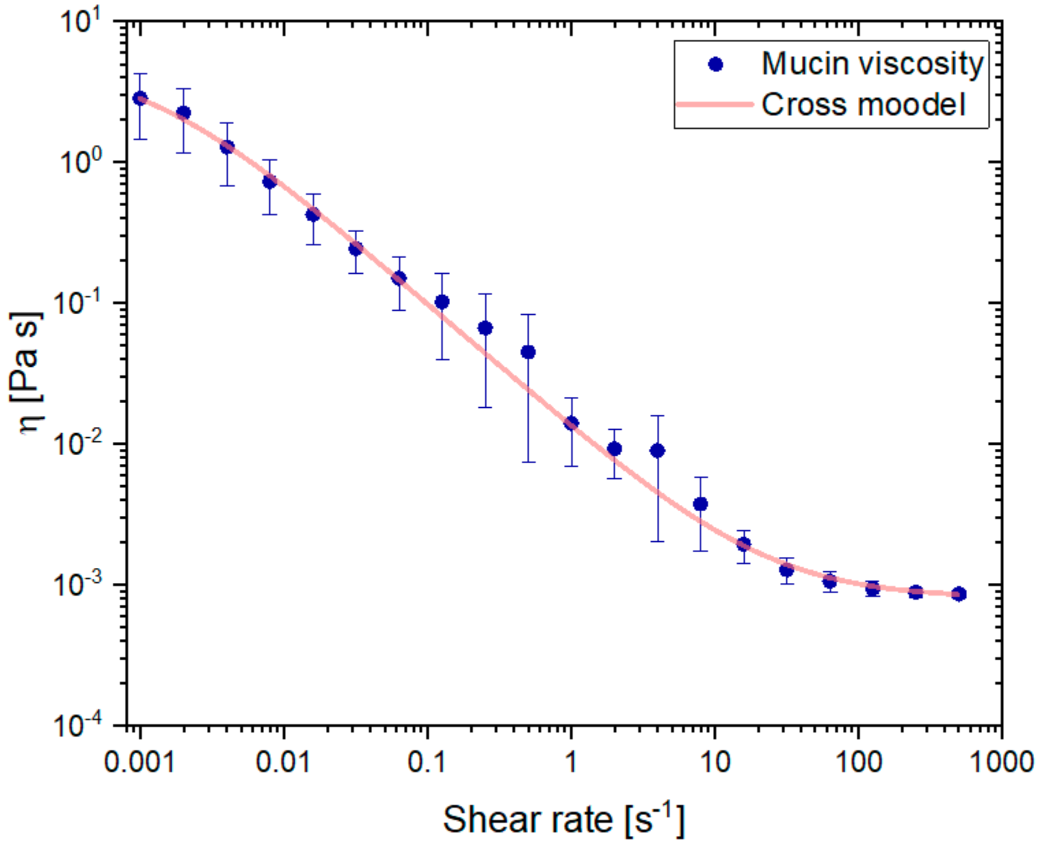

Figure S3. Flow curve of a 1.25 mg/mL1 mucin solution. The viscosity η decreases markedly with increasing shear rate, indicating a pronounced shear-thinning behavior. Experimental data (blue circles) were fitted using the Cross model (redline), yielding a zero-shear viscosity η_0 of approximately 5.32 Pa s and a flow index n around 0.23 in the power-law region.

References

1. Yu, M.; Guo, X.; Lu, H.; Li, P.; Huang, R.; Xu, C.; Gong, X.; Xiao, Y.; Xing, X. Carbon dots derived from folic acid as an ultra-succinct smart antimicrobial nanosystem for selective killing of S. aureus and biofilm eradication. *Carbon* **2022**, *199*, 395-406.
2. Koç, O.m.K.; Üzer, A.e.; Apak, R.a. High quantum yield nitrogen-doped carbon quantum dot-based fluorescent probes for selective sensing of 2, 4, 6-trinitrotoluene. *ACS Applied Nano Materials* **2022**, *5*, 5868-5881.
3. Cao, X.; Bansil, R.; Bhaskar, K.R.; Turner, B.S.; LaMont, J.T.; Niu, N.; Afdhal, N.H. pH-dependent conformational change of gastric mucin leads to sol-gel transition. *Biophysical journal* **1999**, *76*, 1250-1258.
